# Obesity and cancer: Mouse models used in studies

**DOI:** 10.3389/fonc.2023.1125178

**Published:** 2023-03-16

**Authors:** Bo-Tao Zhang, Jia-Ying Xu, Wei Wang, Yang Zeng, Jun Jiang

**Affiliations:** ^1^ Department of General Surgery (Thyroid Surgery), the Affiliated Hospital of Southwest Medical University, Luzhou, China; ^2^ Department of Orthodontic, the Affiliated Stomatological Hospital of Southwest Medical University, Luzhou Key Laboratory of Oral & Maxillofacial Reconstruction and Regeneration, Luzhou, China

**Keywords:** obesity, cancer, diet-induced obesity, mouse model, preclinical disease model

## Abstract

There is increasing evidence that obesity is associated with the occurrence and development of malignant tumors. When studying the relationship between obesity and malignant tumors, it is very important to choose an appropriate animal model. However, BALB/c nude mice and other animals commonly used to study tumor xenograft (human-derived tumor cell lines) transplantation models are difficult to induce obesity, while C57BL/6 mice and other model animals commonly used for obesity research are not suitable for tumor xenograft transplantation. Therefore, it is difficult to replicate both obesity and malignancy in animal models at the same time. This review summarizes several experimental animal models and protocols that can simultaneously induce obesity and tumor xenografts.

## Introduction

Over the past few decades, obesity has become a growing global health problem. From 1975 to 2016, the global prevalence of obesity nearly tripled, affecting 13% of the world’s adult population (1). A large body of epidemiologic evidence shows that obesity is associated with the incidence and progress of several cancers. According to the World Cancer Research Fund’s Third Expert Report, obesity is an important risk factor for many types of cancer (2). The mechanisms linking obesity and cancer development remain unclear. The impact of obesity on human health may take decades to become apparent. Therefore, the use of experimental animals to study the effects of obesity on cancer is of great importance for the discovery of the phenomenon and the study of the mechanism.

Researchers often use preclinical animal models to study the relationship between obesity and disease. Because gene knockout and transgenic technology cannot fully reflect the pathogenesis and pathogenic factors of obesity, the current modeling method is still based on food inducing. Immunodeficient mice are widely used in cancer research. Because xenografts can be performed, they provide researchers with insight into the growth, invasion, and metastasis of human tumor cells. In addition, researchers have also created several types of genetically engineered mouse models (GEMMs) that can spontaneously develop cancer.

However, replicating both obesity and malignancy in laboratory animals is extremely difficult. Animals commonly used in obesity models cannot engraft heterolytic tumors. On the other hand, it is difficult to induce obesity in animals commonly used in cancer models. This situation leaves researchers with limited options. In this review, we discuss the mouse model and related experimental strategies for obesity and cancer research.

## Obesity model

Animal models of obesity are diverse and include both mammalian and nonmammalian species. Non-mammals have certain limitations due to major anatomical and physiological differences from humans (3). Therefore, mammals are usually considered the ideal animal model for obesity research. Among mammals, mice are most used. This is because of their small body size, high reproductive capacity, relatively short life cycle, and relatively easy genome editing (4, 5).

### Diet-induced obesity

Diet-induced obesity (DIO) is an important model of obesity and results from excessive consumption of a high-fat diet (HFD), which usually contains 45-60% fat (6). DIO can simulate the development of human obesity better than genetic models (7, 8) and commonly use the mouse as the model (9). Consumption of HFD can lead to central obesity and insulin resistance in mice and is a good research alternative to mimic diet-induced obesity in humans.

#### Mouse species

Among inbred mice, C57BL/6J, BALB/c, KM, and ICR mice are commonly used to reproduce DIO models (10). Other inbred strains, such as SWR/J and A/J mice, are less sensitive to high-fat diets and related complications (11). The C57BL/6J has the advantage of short modeling time and stable metrics, so it is the most widely used. The C57BL/6J is more susceptible to fat accumulation, weight gain, and glucose metabolism disorders when fed a high-fat diet, as manifested by significant changes in abdominal fat weight, Lee’s index, and adipocyte volume.

#### Age and sex of the mice

The weight of C57BL/6J mice gradually increased with age, reaching the peak at approximately 9 months (12). Compared with the younger mice, the older ones (22 months old or older) had less muscle and more fat (13). Male mice are often used in experimental studies to induce obesity because they are more sensitive to high-fat diets and are prone to diet-induced insulin resistance and abnormal glucose tolerance (14, 15). Compared with male mice, female mice gain weight slowly, have a low obesity rate, and are generally resistant to high-fat diet-induced obesity (16, 17). However, because brown adipose tissue is easier to observe in female mice, female C57BL/6J mice are generally used to study the role of brown adipose tissue in energy metabolism (18).

### Monogenic obesity model

Two types of spontaneously obese mice based on C57BL/6J were identified at the Jackson Laboratory, ob/ob mice in 1950 and db/db mice in 1965. The ob/ob mice lack functional leptin, whereas the db/db mice lack functional leptin receptors. Both types of mice exhibit overeating and are the primary mouse models for studies of monogenic obesity (4, 7). The ob/ob mice have a single base pair mutation in the ob gene, resulting in the absence of functional leptin, increased body weight, hyperphagia, and a low resting metabolic rate. On the other hand, due to a defect in the leptin receptor, leptin signaling is impaired in the db/db mice, resulting in significantly higher serum leptin levels. Therefore, the treatment of reorganization is sufficient to make ob/ob mice normal (19), but it is not effective for db/db mice (20). In addition, the two types of mice are the same in obesity, hypogonadism, and growth hormone (GH) deficiency (4, 6).

Monogenic obesity models have become important research tools in modern drug discovery. The ob/ob mouse is commonly used to evaluate the efficacy of new obesity drugs in overcoming the obesity phenotype caused by overeating (21), and db/db mice are commonly used to study the efficacy of antidiabetic drugs (22). These models require only short-term rather than long-term feeding to induce obesity. However, monogenic models generally do not represent the full pathogenesis of human obesity. Monogenic obesity in humans accounts for only a small proportion of obesity, and a few of human obesity can be explained by mutations in leptin or leptin receptors alone.

### Polygenic obesity model

Compared to the monogenic model, the polygenic model can better simulate the pathogenesis of human obesity. The C57BL/6J mouse is the most used obese mouse model, which is susceptible to obesity induced by overeating. However, only 60% of C57BL/6J mice gain weight under high-fat diet conditions. The susceptibility of C57BL/6J mice to diet-induced obesity is typically characterized by changes in plasma insulin and leptin levels and insulin sensitivity at 6 weeks of age (23). New Zealand obese (NZO) mice are polygenic inbred mice predisposed to obesity and type 2 diabetes. Unlike C57BL/6J mice, NZO mice can gain weight on a standard diet (24).

## Tumor mouse model

Mice have similar biological, physiological and pathological characteristics to humans and exhibit a high degree of genetic similarity, making them an ideal animal model for the study of tumors. Much of the current understanding of human cancer characteristics are based on long-term *in vitro* culture of tumor cell lines and their inoculation into mice.

### Tumor implantation model

Currently, most tumor implantation models used in basic or translational oncology research are based on established cell lines (25). They usually function as allografts of primary mouse tumors or xenografts of human tumors. In both types of models, cancer cells can be injected orthotopically or ectopically (mainly subcutaneously) and subsequently monitored for growth or metastasis (intraperitoneally, intravenously, or intracardially).

Since 1950, allografts have been used primarily as a preclinical model for drug development and cancer therapy (26). For example, researchers established the leukemia model using male DBA/2 mice and found that AZD2014, an mTORC1/2 inhibitor, inhibited the growth and proliferation of L1210 leukemia cells (27). The toxicology of some cytotoxic drugs has also been successfully studied in allograft models. However, allograft tumor models are of limited value for the study of human tumors. Therefore, xenograft tumor models have replaced allograft tumor models as the primary tool for preclinical drug testing since 1990.

#### Tumor ectopic transplantation model

The discovery of the thymus-free nude mouse was a major breakthrough in cancer research, allowing human tumors to be replicated in xenogeneic experimental animals. Immunodeficient mice have remarkable xenograft success rates and are able to preserve the original tissue structure and function of human cancers. Representative immunodeficient mice include nude mice, severe combined immunodeficiency (SCID) mice, non-obese diabetic/SCID (NOD/SCID) mice, and NOD-SCID-IL2Rg^-/-^ (NSG) mice (**Table 1**). SCID mice have been shown to be more suitable for human cancer cell xenografts than nude mice and have advantages for studying the biology of human tumors *in vivo* and their response to therapy (28).

**Table 1:** Characteristics and application of common immunodeficient mice.

Mouse strains	Background	Characteristic	Application	Notes
Nude	BALB/c	Mutations in the Foxn1 gene result in thymic aplasia, lack of T cells, and no immunological rejection.	It plays an important role in tumor, immunity, drug safety evaluation and preclinical screening of drugs.	It is not suitable as a host for leukemia or lymphoma because human hematopoietic stem cells are not transplantable into nude mice.
CBA/N	CBA/H	Btk gene mutations, defective B lymphocyte function, absent humoral response.	It can be used in the bone marrow transplantation model and is an ideal tool for studying the production, function and heterogeneity of B lymphocytes.	The incidence of spontaneous tumors is low and rarely used in oncology studies.
Beige	C57BL/6	Beige gene mutations, defective NK cell development and function and impaired humoral response.	It is widely used in immunology research.	It is more sensitive to various pathogenic factors and needs a good SPF environment.
SCID	CB-17	Mutations in the Prkdc^scid^ gene result in V(D)J recombination *in vivo* and defects in the generation of T and B-cell.	It is a good candidate for the xenograft tumor, especially blood-derived tumor cells and initially used as recipients of human hematopoietic stem cell and peripheral blood mononuclear cell transplantation.	SCID mice are more prone to die from infections. Among a small number of SCID mice, a certain degree of immune recovery may occur in young adulthood.
NOD/SCID	NOD	Prkdcscid gene mutation in NOD background. In innate and adaptive immune deficiency, various tumor cells can be implanted, with less rejection and graft-versus-host disease.	NOD-SCID mice accept allogeneic and xenogeneic grafts, making them a suitable model for cell transfer experiments. A high degree of immunodeficiency but low immune infiltration.	Spontaneous thymic lymphoma occurs, resulting in a shorter lifespan, which makes it unsuitable for long-term transplantation.
NRG	NOD	NOD background carrying Rag1^null^ and IL2rg^null^ gene mutations. Deficient in B, T and NK T cells.	Human hematopoietic stem cells containing CD34+ and PDX can be efficiently transplanted to establish transplanted humanized mice models.	More resistant to irradiation and genotoxic agents than Prkdcscid mice.
NSG	NOD	NOD background carrying Prkdc^scid^ and IL2rg^null^ gene mutations. Deficient in B, T and NK T cells.	It is widely used in humanized mouse models of immunology, drugs, viruses and tumors.	An internationally recognized animal model with the highest degree of immunodeficiency and most suitable for xenotransplantation. Low incidence of lymphoma, low immune infiltration, sensitivity to radiation.
BRG	BALB/c	BALB/c background carrying Rag1^null^ and IL2rg^null^ gene mutations. Deficient in B, T and NK T cells.	It is a super immunodeficient mouse that is useful for research on humanization, infectious diseases, autoimmune diseases and in xenograft assays.	May be an ideal animal model to replace SCID mice in the future.

Nude, Nude mice; PDX, Patient-Derived Xenograft; NK cell, Natural Killer cell; NOD, Non-Obese Diabetes; SCID, Severe Combined Immunodeficiency; NRG, NOD-Rag1^-/-^-IL2rg^-/-^; NSG, NOD-SCID-IL2rg^-/-^; BRG, BALB/c-Rag1^-/-^-IL2rg^-/-^.

Ectopic transplantation typically inoculates human cancer cell lines or pieces of tumor tissue under the skin in the axilla, back and hind legs of mice. After subcutaneous inoculation, the tumor tissue is surrounded by a thick fibrous capsule and rarely metastasizes to adjacent tissues. Tumor growth can be easily observed and the treatment efficacy can be evaluated.

#### Tumor orthotopic transplantation model

The microenvironment of *in situ* implanted tumors is different from that of ectopically implanted tumors, and therefore their growth rates are different. Because growing in an optimal microenvironment, *in situ* implanted tumors generally exhibit more active proliferation, metastasis, and invasion, which better mimics the growth of tumors in the human body (29). Fu XY et al. orthotopically implanted human colon cancer cells in the colon of nude mice. The transplanted tumors almost exactly replicated the characteristics of the corresponding human cancer, which included local tumor growth, abdominal metastasis with peritoneal seeding, liver metastasis, lymph node metastasis, and intestinal obstruction (30). Carmelo Nucera et al. established an orthotopic model of human thyroid cancer using the anaplastic thyroid carcinoma cell line 8305C and observed tumor growth and metastasis (31). However, because the volume and number of tumors in the visceral organs are not easily measured, there are cases where tumor ectopic transplantation is more appropriate.

#### Tumor intravenous transplantation model

The above ectopic and orthotopic transplantation models, also called spontaneous tumor metastasis model. The method of injecting cancer cells directly into the blood to study their spread and metastasis is called experimental tumor metastasis model. The experimental metastasis model is used to study the growth of malignant tumors in distant organs. Intravenous injection can shorten the time of tumor formation in target organs. Inoculation *via* the tail vein is one of the most used methods in the experimental metastasis model. For example, Nan Huo et al. established a lung metastasis model for thyroid cancer by injecting TPC-1 cells into BALB/c nude mice *via* the tail vein (32).

### Genetically engineered mouse model

In the 1980s, the development of transgenic and gene-targeting technologies in mouse embryonic stem cells facilitated the generation of GEMM. The most common ways to generate GEMM are to activate oncogenes or inactivate tumor-suppressor genes *in vivo* through the use of transgenic and gene targeting methods, such as knock-outs and knock-ins. Gordon et al. established the first transgenic mice in 1980, harboring randomly integrated oncogenes under the control of a tissue-specific promoter (33). The initial set of genetic engineering tools was set against the background of the emergence of genome-editing technologies such as restriction endonucleases, DNA cloning and sequencing, and then developed lentiviral vector, electrotransfection and microinjection techniques. In 2016, the single-base gene editing technology developed from CRISPR-Cas9 avoided DNA double-strand breaks and further expanded the scope of base editing. The innovation of gene editing technologies has significantly reduced the time needed to establish a GEMM (34). GEMM has been used in the study of colorectal cancer (35), renal cell carcinoma (36) and breast cancer (37). In addition, it can be used in preclinical trials for hormonal and targeted therapies as well as immunotherapy. PD-1 KO and PD-L1 KO mice have been exploited to develop drugs for cancer treatment (38).

## Obesity-associated cancer model

*In vivo* animal models are important research tools to study the underlying mechanisms of the association between obesity and cancers. Among genetic models of obesity, mice deficient in leptin signaling are the most used. When mice were fed standard chow, the genetic model showed early-onset obesity and comorbid diseases such as insulin resistance and hepatic steatosis. Their main disadvantage is the exclusion of the factors other than leptin that may affect cancer cells and tumor microenvironment. For example, obesity accelerates the progress of Kras-driven pancreatic ductal adenocarcinoma, but not lung cancer (39).

The DIO mouse model is believed to mimic human obesity well and to explain the potential biological link between obesity and cancer. The DIO model was established by feeding mice a diet high in sugar, fat or both. While several feeding regimens have been developed, the most commonly used diets contain 30% to 60% kcal from fat, which is fed to the mice for 10 to 12 weeks prior to tumor formation.

Most obesity-related complications are due to inflammation (40). Chronic inflammation in adipose tissue, especially white adipose tissue (WAT), stimulates cancer progression through mechanisms such as altered levels of adipokines and inflammatory mediators, and insulin resistance (41–43). Short-term HFD feeding is difficult to obtain an ideal model sufficient to study the relationship between obesity and cancer (44). Therefore, long-term obesity models need to be established to simulate the relationship between human obesity and tumors. The feeding time of the HFD-induced obesity mouse model ranged from 4 weeks to 56 weeks, and 10 weeks to 12 weeks were usually selected (**Table 2**). DIO mice gain weight, increase fasting blood glucose levels, and develop obesity-related phenotypes such as hyperinsulinemia, insulin resistance, hepatic steatosis, hypertension, and dyslipidemia (59). Whether reversal of the obesity phenotype affects tumor prognosis is a key question in this field. Dietary pattern switching experiments have shown that once DIO is established, a low-fat diet (LFD) for a prolonged period, such as 5 weeks, is sufficient to reverse obesity-induced chronic inflammation and tumor progression (44, 52).

**Table 2:** Overview of obesity-associated cancer model.

Obesity model	Mouse strains	Diet	Duration	Cancer model	Obese tumor phenotype/proposed mechanism	Ref
DIO	Nude	HFD (35% kcal from fat)	4 weeks	T (TE-1, 2.0×106 cells, subcutaneous)	Obesity Potentiates Esophageal Squamous Cell Carcinoma Growth and Invasion by AMPK-YAP Pathway	(45)
		HFD (40% kcal from fat)	16 weeks	T (SKOV3i.p-RPF, 5×10^6^ cells in 2ml PBS, orthotopic)	Obesity Contributes to Ovarian Cancer Metastatic Success Through Increased Lipogenesis, Enhanced Vascularity, and Decreased Infiltration of M1 Macrophages	(46)
		HFD	16 weeks	T (PC3.pGIPZ/PC3.shCtBP1, 4.8 × 10^6^ cells, subcutaneous)	Prostate Tumor Growth Is Impaired by CtBP1 Depletion in High-Fat Diet–Fed Mice	(47)
	C57BL/6	HFD (42% kcal from fat)	4 months	–	High fat diet promotes prostatic basal-to-luminal differentiation and accelerates initiation of prostate epithelial hyperplasia originated from basal cells	(48)
		HFD (42% kcal from fat)	40 weeks	GEMM (Alb-Cre; Ptpn2fl/fl)	Obesity Drives STAT-1-Dependent NASH and STAT-3-Dependent HCC	(49)
		HFD (60% kcal from fat)	Until endpoint	GEMM (MUP-uPA)	Endoplasmic reticulum stress cooperates with hypernutrition to trigger TNF-dependent spontaneous HCC development	(50)
			8 weeks	T (AsPC-1, 1×10^5^ cells, orthotopic) GEMM (KPC mice)	Critical role for arginase 2 in obesity-associated pancreatic cancer	(51)
			9-14 months	T (Apc-null Lgr5-GFP^hi^ ISCs/Lgr5-GFP^low^ progenitors cells, orthotopic)	High fat diet enhances stemness and tumorigenicity of intestinal progenitors	(52)
	NSG	HFD (60% kcal from fat)	7days	T (SCC-25, FaDu, Detroit-562, JHU-029, orthotopic)	Targeting metastasis-initiating cells through the fatty acid receptor CD36	(53)
	-	HFD (60% kcal from fat)	From 8 weeks until endpoint	GEMM (ThrbPV/+Pten+/−)	Inhibition of STAT3 activity delays obesity-induced thyroid carcinogenesis in a mouse model	(54)
			From 6 weeks until endpoint	GEMM (ThrbPV/+Pten+/−)	Diet-induced obesity increases tumor growth and promotes anaplastic change in thyroid cancer in a mouse model	(55)
	BALB/c	HFD (60% kcal from fat)	20 weeks	T (CRL-2947-Luc, orthotopic)	Elevated Leptin during Diet-Induced Obesity Reduces the Efficacy of Tumor Immunotherapy.	(56)
MOM	ob/ob	–	–			
DIO	C57BL/6	HFD (60% kcal from fat)	10 weeks	T (Pan02, AK4.4, orthotopic)	Obesity-induced inflammation and desmoplasia promote pancreatic cancer progression and resistance to chemotherapy	(57)
MOM	ob/ob	–	–			
	ob/ob db/db	–	–	T (Pan02, 2.5×10^5^, subcutaneous)	Obesity potentiates the growth and dissemination of pancreatic cancer	(58)
	ob/ob db/db	–	–	GEMM (KC crossed with ob/ob)	Endocrine-Exocrine Signaling Drives Obesity-Associated Pancreatic Ductal Adenocarcinoma.	(39)

Nude, nude mice; HFD, high fat diet; T, transplant model; GEMM, genetically engineered mouse model; ISCs, intestinal stem cells; MOM, monogenic obesity model (unless otherwise listed, duration of feeding indicates feeding pattern before transplantation or induction of cancer).

Nude mice used to establish tumor xenograft models, such as BALB/c, are generally difficult to induce obesity. Stemmer K et al. found that Foxn1 nude mice (B6. Cg-Foxn1nu/J) on a C57BL/6 background fed a high-fat diet under thermoneutral (33°C) conditions significantly increased their body weight (60), making them an excellent model for studying obesity and tumors.

## Discussion

Obesity is an important risk factor for cancer. Significant attention has been paid to the underlying mechanism between the two diseases. Appropriate animal models replicating both obesity and cancer are highly needed to study their association. A brief review shows that there is currently no single ideal model for this type of research (**Table 2**). The models listed are good for studying tumor progression and metastasis, but there are also some shortcomings. They cannot determine how diet and obesity contribute to cancer initiation and be used to study cancer survivorship.

The mouse models utilize high-fat diets to achieve obese condition but the typical western diet that is most closely associated with obesity and cancer is composed of a dietary pattern comprised of high protein and fat but most importantly very high in refined sugars (61, 62). This particular dietary pattern is not similar to mouse models and although it would be difficult to replicate in models the shortcomings should be noted (63). Humans who are exposed to high carbohydrate diets will not only lead to weight gain and obesity, but exacerbate glucose/insulin homeostasis which could be an important underlying mechanism associated with the progression of cancer independent of obesity or perhaps in synergy (64). Furthermore, a western dietary pattern has been associated with inflammation (65–67) and this is another important exposure that is missing in most animal models of cancer.

When selecting an appropriate mouse model, factors such as obese phenotype, environmental stimuli, mouse strain and sex should be considered more fully. With the development of different mouse models, the combined application of multiple models makes cancer research more convenient and accurate. Recently, the emergence of a revolutionary CRISPR/Cas9 system has greatly enhanced the efficiency of precise gene editing in various GEMMs. However, the potential risk of off-target effects is a notable concern. An ideal cancer + obesity mouse model should be technically simple, quick in operation, easily reproducible, affordable and short in modeling. Further improvement of obesity-prone mice that can be implanted with human tumor cells will help decipher the mechanism by which obesity affects tumor initiation and progression.

## Author contributions

JJ and YZ contributed the central idea and analyzed most of the data. B-TZ and J-YX wrote the initial draft of the paper, WW contributed to refining the ideas and carrying out additional analyses. All authors reviewed the manuscript. All authors contributed to the article and approved the submitted version.
